# Metacognitive therapy versus exposure and response prevention for obsessive-compulsive disorder: study protocol for a randomized controlled trial

**DOI:** 10.1186/s13063-019-3381-9

**Published:** 2019-05-20

**Authors:** Kim Melchior, Ingmar Franken, Mathijs Deen, Colin van der Heiden

**Affiliations:** 1 Outpatient Treatment Centre PsyQ & Erasmus University Rotterdam, Max Euwelaan 70, 3062 MA Rotterdam, the Netherlands; 2 Erasmus University Rotterdam, Burgermeester Oudlaan, 3062 PA Rotterdam, the Netherlands; 3 Parnassia Psychiatric Institute & Leiden University, Monsterseweg 83, 2553 RJ Den Haag, the Netherlands

**Keywords:** Obsessive-compulsive disorder, Metacognitive therapy, Exposure and response prevention, Randomized controlled trial

## Abstract

**Background:**

The recommended psychological treatment of choice for obsessive-compulsive disorder (OCD) is exposure with response prevention (ERP). However, recovery rates are relatively modest, so better treatments are needed. This superiority study aims to explore the relative efficacy of metacognitive therapy (MCT), a new form of cognitive therapy based on the metacognitive model of OCD.

**Design and method:**

In a randomized controlled trial, we will compare MCT with ERP. One hundred patients diagnosed with OCD will be recruited in an outpatient mental health center in Rotterdam (the Netherlands). The primary outcome measure is OCD severity, measured by the Yale-Brown Obsessive Compulsive Scale (Y-BOCS). Data are assessed at baseline, after treatment, and at 6 and 30 months follow-up.

**Discussion:**

By comparing MCT with ERP we hope to provide an indication whether MCT is efficacious in the treatment of OCD and, if so, whether it has the potential to be more efficacious than the current “gold standard” psychological treatment for OCD, ERP.

**Trial registration:**

Dutch Trial Register, NTR4855. Registered on 21 October 2014.

**Electronic supplementary material:**

The online version of this article (10.1186/s13063-019-3381-9) contains supplementary material, which is available to authorized users.

## Background

### Phenomenology and treatment

Obsessive-compulsive disorder (OCD) is a severe mental condition which is characterized by intrusive thoughts (obsessions) and repetitive behaviors (compulsions) intended to neutralize anxiety induced by these thoughts [1]. OCD has been ranked among the 10 most debilitating disorders by the World Health Organization (WHO) and tends to be chronic without adequate treatment [48]. Both studies into pharmacological treatment, primarily with selective serotonin reuptake inhibitors (SSRIs), and studies into specific forms of psychological treatment supported the effectiveness of these treatment modalities in reducing symptoms of OCD [3]. The first-choice psychological treatment for OCD is exposure and response prevention (ERP) [31, 34, 37], a specific type of cognitive behavioral therapy (CBT) based on learning theory, which suggests that classical conditioning is responsible for the development of obsessions, whereas operant conditioning processes maintain anxiety and compulsive behaviors [27]. In ERP treatment, patients are exposed to anxiety-provoking stimuli (situations, objects, thoughts) combined with the strict prevention of performing ritual behaviors [26]. Since its introduction in 1966, the prognosis for OCD improved substantially. However, OCD remains a difficult disorder to treat. Although numerous studies have found statistically significant change and large improvements in OCD symptoms after ERP, the outcomes are sub-optimal for the majority of patients. More specifically: although about 60% of treatment completers achieve recovery, only approximately 25% of patients are asymptomatic following treatment [11, 14], which means that the majority of patients treated with ERP continue to experience distressing OCD symptoms. Furthermore, the overall effectiveness of ERP for OCD is attenuated by some limitations of the approach. As approximately 30% of patients with OCD refuse ERP or drop out from treatment prematurely, it is assumed that overall recovery rates are lower [30]. Moreover, these figures suggest that ERP might be hard to tolerate and is burdensome, which is supported by the finding that an important reason for not attempting ERP are the requirements of treatment (e.g. exposure to anxiety provoking stimuli [46]). So, although it can be concluded that ERP is efficacious, there is clearly room for improvement in the psychological treatment of OCD. It is assumed that this improvement could result from a better understanding in the mechanisms involved in the maintenance of the disorder.

### The metacognitive model of OCD

**Fig. 1:**
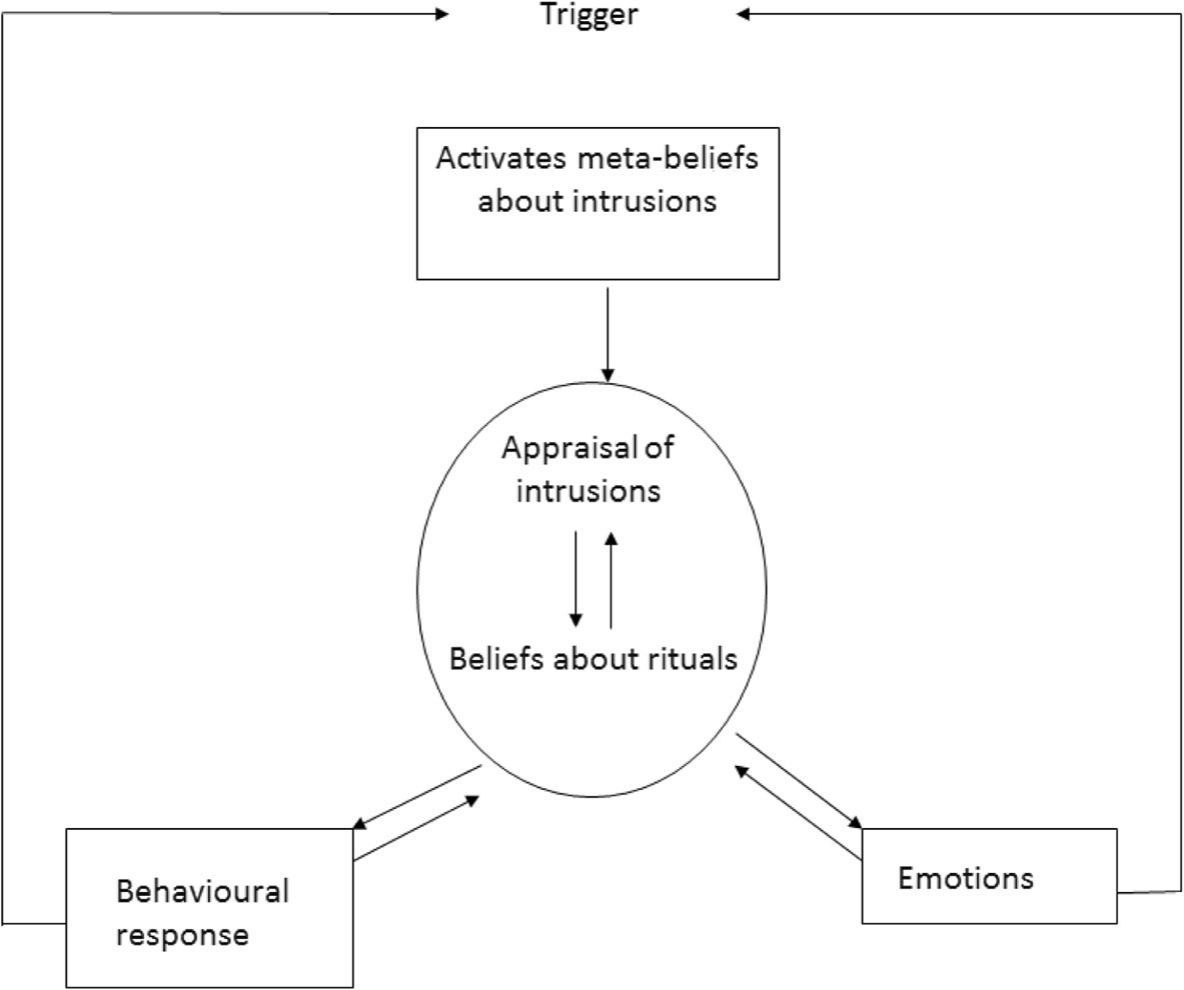
Metacognitive model for OCD [42]

A recently developed theoretical account explaining the maintenance of OCD symptoms is the metacognitive model by Adrian Wells [42, 43]. In this model of OCD, two belief domains are assumed to be fundamental in the maintenance of the disorder. First, it is proposed that obsessions are misinterpreted because of metacognitive beliefs about the dangerousness, significance, and consequences of intrusive thoughts and feelings, the so-called fusion beliefs. Three classes of fusion beliefs are highlighted: thought action fusion (TAF); thought event fusion (TEF); and thought object fusion (TOF). TAF [32] refers to the belief that obsessional thoughts can lead to the commission of an action (e.g. “thinking about killing someone will make me do it”). TEF [42] refers to the belief that obsessional thoughts can make events happen (e.g. “thinking about a car accident means I will be involved in such an accident”) or mean an event has already occurred (e.g. “If I think I ran into someone with my car, I probably did it”). Finally, TOF [43] refers to the belief that thoughts or negative feelings can be passed into objects (e.g. “my feeling of evil could be passed into objects and from these objects to other people”). Once the fusion beliefs are activated, they give significance to obsessional thoughts and lead to appraisal of, and worrying about, the thoughts and consequently to feelings of anxiety and perceived threat. This anxiety primes a second domain of metacognitive beliefs: beliefs about the necessity of performing rituals in response to obsessive thoughts in order to reduce the perceived threat (e.g. “Counting to seven will restrain me from acting on my thoughts”). Consequently, patients with OCD engage in both overt and covert ritual behaviors and, thereby, use specific internal rules (instead of external observation) and so-called “stop signals” to determine how the ritual must be conducted and when it can be terminated. Such stop signals are often metacognitive experiences, such as a feeling of satisfaction (e.g. “I must wash my hands until ‘it feels right’”). They also use other neutralizing coping strategies such as monitoring for further intrusive experiences, which is seen as a counterproductive strategy as it increases the awareness and frequency of intrusive thoughts. The metacognitive model of OCD is illustrated in Fig. 1.

### Metacognitive treatment for OCD

Based on the metacognitive model, treatment should focus exclusively on modifying patients’ beliefs about the importance and power of intrusive thoughts and the necessity of performing rituals, instead of challenging the actual content of the obsessions and compulsions [12]. Although metacognitive therapy (MCT) uses comparable techniques as cognitive therapy (CT) for this purpose, such as verbal reattribution and behavioral experiments, the two approaches are fundamentally different [13]. For example, patients with OCD can describe appraisals in the domain of inflated responsibility, perfectionism, and intolerance of uncertainty. The metacognitive model proposes that such appraisals result from the activation of metacognitive beliefs about obsessions; consequently, it is not necessary to modify these lower order beliefs as is done in CT (e.g. by using the pie chart technique to compare the patient’s original estimated probability with a more realistic estimate of probability) [5, 17]. Targeting such lower order beliefs and automatic thoughts is seen as counterproductive as it just promotes further conceptual processing, such as worrying and rumination [15]. Instead, it is thought that modification of the metacognitive beliefs about the meaning and power of obsessions removes the need for further conceptual processing. Therefore, interventions are explicitly aimed at the metacognitive processes which perpetuate the continued maladaptive processing instead of attempting to modify the content of perseverative thinking (i.e. appraisals) [13].

So far, there is preliminary evidence supporting the efficacy of MCT for OCD. The clinical significance of treatment effects in the following mentioned studies is calculated using the standard criteria developed by Fisher and Wells [11, 14], based on the method of Jacobson and Truax [21]. Based on these criteria, patients are classified as recovered if they achieved a reduction of minimal 10 points on the Yale-Brown Obsessive Compulsive Scale (Y-BOCS [19]; a semi-structured interview for OCS) and a post-treatment score < 14. When achieving a post-treatment score < 7, patients are classified as asymptomatic. Using single case methodology, Fisher and Wells [12] found clinically significant improvements for four OCD patients with different clinical presentations who were treated individually with MCT. Two of the four participants were asymptomatic at both post-treatment and three-monthfollow-up assessments. Furthermore, Rees and Van Koesveld [33] found that seven out of eight participants in an open trial of group MCT for OCD reached criteria for a recovery status on the Y-BOCS at three-monthfollow-up (87.5%). In an additional study, Fitt and Rees [16] found similar clinically significant reductions among three patients treated with MCT using videoconference. In an open trial of individual metacognitive therapy among 25 patients with OCD, Van der Heiden et al. [39] found statistically significant reductions on all outcome variables. Moreover, in terms of clinically significant results, 74% of the treatment completers (*n* = 19) were classified as recovered after treatment and 47% as asymptomatic. At follow-up, this increased to 80% and 67% respectively. Finally, Simons et al. [35] found positive outcomes of MCT in comparison to ERP in the treatment of pediatric OCD in a case series design. Together, these findings suggest that MCT might be an efficacious treatment for OCD and deserves controlled evaluation. The present trial has been initiated to compare the relative efficacy of MCT with ERP, in an outpatient clinical sample of patients with OCD. Our main hypothesis is that MCT is more efficacious than ERP in the treatment of OCD in terms of both statistically and clinically significant improvements, both directly after treatment (primary outcome) and at follow-up.

## Design and methods

### Design

**Fig. 2:**
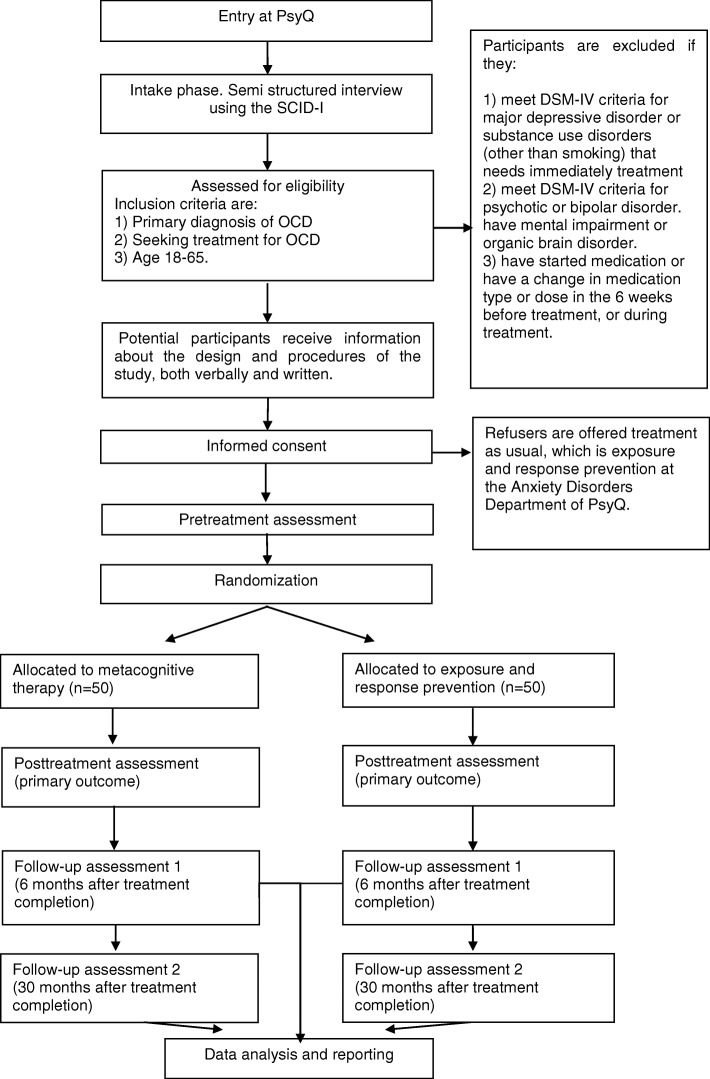
*Flowchart* of the study

**Fig. 3:**
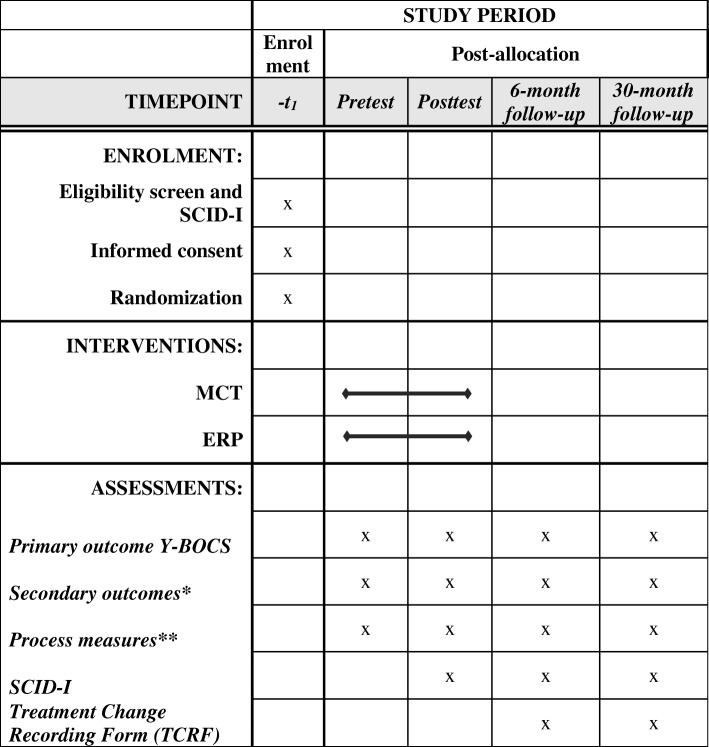
Standard protocol Items: Recommendations for Interventional Trials (SPIRIT). Diagram of enrolment, interventions and assessments over time. MCT metacognitive therapy, ERP exposure and response prevention, SCID-I Structured Clinical Interview for DSM Axis I Disorders. Primary outcome measure: Yale-Brown Obsessive Compulsive Scale (Y-BOCS). *Secondary outcomes: Padua Inventory-Revised (Padua IR); The Symptom Checklist (SCL-90); The Beck Depression Inventory, 2nd version (BDI-II); World Health Organization Quality of Life (WHOQOL-Bref); Obsessive Belief Questionnaire (OBQ-44). **Process measures: Thought Fusion Instrument (TFI); and Beliefs About Rituals Inventory (BARI)

We will conduct a randomized controlled trial (RCT) with a pretest–post-test (primary outcome) 6-month–30-month follow-up-design. Patients will be recruited from consecutive referrals to the Anxiety Disorders department of PsyQ, an outpatient community mental health center in the Netherlands. After screening for eligibility and informed consent, we will randomize the patients into two groups: MCT and ERP. The number of excluded patients and refusers and their reasons are registered. Participating patients will be assessed by self-report measures and semi-structured clinical interviews administered by a research assistant who is blind to group allocation at entry (pre-treatment), after the last treatment session (post-treatment – primary outcome), six months (first follow-up) after treatment completion and 30 months (second follow-up) after treatment has ended. The latter assessment is included to answer a secondary research questions on the durability of both the ERP and MCT effect on the long term. Due to a lack of studies with follow-up periods of > 1 year [9, 47], the information on longer-term effects are unknown. In case of drop-out, measurements and interviews are also administered directly after treatment had ended whenever this is possible. The study has received ethical approval from the Medical Ethical Committee of the Leiden University Medical Centre (LUMC) (protocol number NL50201.058.14) and is registered in the Dutch Trial Register (protocol number NTR4855). All data will be stored anonymously; there is a data safety and monitoring board for the study. Figure 2 shows a flowchart of the study from patient enrollment up to data analysis and reporting. This study follows the “guidance of standard protocol items: recommendations of interventional study’s (SPIRIT).” The SPIRIT figure template is displayed in Fig. 3. In addition, the SPIRIT checklist can be found in Additional file 1.

### Sample size

There are no studies available directly comparing ERP with MCT. We chose to design our study with enough statistical power to enable us to detect a medium between-group effect (Cohen’s *d* = 0.5 [6]) from baseline to post-treatment. We chose for this medium between-group effect because expecting a larger difference between the two treatment groups does seem unrealistic since numerous studies have found statistically significant change and large improvements in OCD symptoms after ERP. On the other hand, designing our study to enable us to detect a small between-group effect is of less relevance for clinical practice. We used the statistical method presented by Liu and Liang [22] for sample size calculations for studies with correlated observations. To achieve a power of 0.80 with four measurement points with a correlation of 0.5 between repeated measures (standard value) and to detect a medium effect size (Cohen’s *d* = 0.50) between the two treatment conditions over time on the primary outcome measure, severity of OCD symptoms, and an expected drop-out rate of 20%, the minimal sample size necessary in each condition is 50.

### Participant enrollment and randomization

One hundred adult patients (aged 18–65 years) with a primary diagnosis of OCD will be recruited from consecutive referrals to the Anxiety Disorders department of PsyQ, an outpatient community mental health center in the Netherlands. Diagnosis of OCD will be established using the SCID-I [10], a diagnostic interview based on the DSM-IV, because diagnostic instruments based on the DSM-5 [1] were not yet available at the development phase of this study. To enhance the clinical representativeness of the sample, exclusion criteria are kept to a minimum. Patients are only excluded if they currently: (1) meet DSM-IV-TR criteria for severe major depressive disorder or substance use disorder (other than smoking) that requires immediate treatment, psychotic disorder, or bipolar disorder; (2) have mental impairment or an organic brain disorder; or (3) had a change in medication type or dose in the six weeks before assessment or during treatment (see Fig. 2). The presence of other co-morbid diagnosis or previous treatment for OCD are not exclusion criteria. Potential participants will receive extensive information about the design and procedures of the study at the end of the clinical screening. Following informed consent, patients will be randomly assigned to the MCT or ERP condition. Randomization will be done by using www.randomization.com, an online generator which randomizes each individual to a treatment condition by using the method of randomly permuted blocks [24]. With randomly permuted blocks, participants are assigned to a treatment condition in blocks to ensure that equal numbers of individuals have been assigned to each treatment, not only at the end of the study but also at various intermediate time points. The generator also randomizes the block sizes (range = 1–4 per group), to ensure that it is unknown when a block is finished; it is not possible to guess the remaining treatment allocation. The process of allocation of cases to intervention conditions will be done by an independent employee of the participating mental health care center using the generated randomization plan. Patients will be allocated to treatment conditions in order of entry. The investigators and therapists have no insight in the randomization plan.

To control for therapist effects, all therapists will deliver both treatments in blocks but not in parallel. For this reason, in the first two years of the study, half of the therapists from each site deliver MCT while the other half does ERP. Two years later, treatment conditions will be crossed over.

### Outcome measures

On all assessment points, the Dutch versions of the measures described below are included. The administration of the SCID-I and the Y-BOCS during intake will be conducted face-to-face and later assessments are by telephone. Self-report measures will be conducted on paper and are home-based.

#### Primary outcome

The primary outcome of interest for this MCT superiority study is OCD severity at post-treatment, which will be measured with the Y-BOCS [19], a semi-structured interview and the “gold standard” for measuring OCD symptoms. The Y-BOCS is a clinician-ratedsemi-structured interview designed to rate the severity of both obsessions and compulsion. The Y-BOCS consists of 10 items rated 0–4 (range = 0–40). The Y-BOCS has been shown to have good psychometric properties and is sensitive for measuring treatment effects [40]. Further, good internal consistency for both the subscales (obsessions and compulsions) and for the total score of the Y-BOCS has been reported [18].

#### Secondary outcomes

The presence of OCD and co-morbid Axis I diagnosis will be assessed with the Structured Clinical Interview for DSM Axis I Disorders (SCID-I [10]). A recently conducted study with a large sample size (*n* = 151) found adequate to good inter-rater reliability for all Axis I disorders [23]. Secondary outcomes include self-report questionnaires to assess OCD symptoms, co-morbid symptoms, and degree of perceived wellbeing. The Padua-Inventory revised (Padua-IR; [4]) is a self-reportmeasure for OCD severity which consists of 60 items scores on a 0–4 scale (range = 0–240). The Padua-IR has reasonable psychometric properties [40]. The symptom checklist (SCL-90 [8]) is used as a measurement of general psychopathology. The SCL-90 consist of 90 items, all scores from 1 (not at all) to 5 (very much; range = 90–450). This self-report measure has shown good psychometric properties. The Beck Depression Inventory, 2nd version (BDI-II [2]) is included to assess the affective, behavioral, somatic, and motivational components of depression. This frequently used self-report consist of 21 items in the range of 1–4 and has good psychometric properties. Finally, World Health Organization Quality of Life (WHOQOL-Bref [48]) is included and assesses the individuals perception of quality of life with respect to physical health, psychological health, social relationships, and environment. The WHOQOL consists of 26 items which are answered on a 5-point scale. It is concluded that the psychometric properties of this questionnaire are good.

#### Process measures

Changes in both belief domains that have been proposed to be important in the etiology of OCD are assessed. To study changes in metacognitive beliefs about the meaning, significance, and danger of intrusive thoughts, the Thought Fusion Instrument (TFI [45]) will be employed. To study changes in metacognitive beliefs about the necessity of performing rituals in response to obsessions, the Beliefs About Rituals Inventory (BARI [25]) is used. The TFI consists of 14 items and the BARI of 12 items. All items are answered on a 4-point scale in the range of 1–4. There are few data available about the psychometric properties of the TFI and the BARI. Gwilliam et al. [20] found reasonable internal consistency, a moderate test–retest reliability, and some support for the convergent and divergent validity for the TFI. In the developmental phase of this study, the psychometric properties of the TFI and the BARI will be further assessed by our research group.

The Obsessive Belief Questionnaire (OBQ-44 [29]) is included as another measurement with the purpose of the assessment of beliefs considered to be important in the maintenance of OCD. Factor analysis of the scale reveals four factors: (1) perfectionism and intolerance of uncertainty; (2) importance and control of thoughts; (3) responsibility; and (4) overestimation of treat [28]. The OBQ-44 consists of 44 items answered on a scale of 1–4. The psychometric properties are good.

In addition, on both follow-up assessments, participants will be called by a research assistant, who will ask them to provide responses for the Treatment Change Recording Form (TCRF [38]), which will be used to assess the initiation, termination, or change of any form of therapy, hospital services, support group, self-help program, or medication utilized by the participant since post-treatment.

### Interventions

The interventions will be offered at the Anxiety Disorders Department of PsyQ, at which ERP is already delivered as treatment as usual for OCD. Both manual-driven treatments consist of up to 15 weekly sessions of 45 min duration. Treatment can be terminated earlier, when both patient and therapist agree that treatment goals are completed. A minimum of eight sessions will be managed as criteria for each patient to can be classified as treatment completer in the statistical analysis. Interventions will be delivered by nine staff psychologists, who are trained in CBT and who are familiar with the provision of ERP for OCD. Four of the participating therapists were trained by Dr. Adrian Wells and Dr. Peter Fisher, experts in the field of MCT, preceding the start of a pilot study into the efficacy of MCT for OCD in which they participated as therapists [39]. The other five therapists will be trained in the provision of MCT for OCD by the fourth author (CH) preceding the start of this study. During the study, therapists will be supervised monthly by the fourth author (CH) in separate group sessions for ERP and MCT. In these 1-h supervision meetings, all current cases and therapy notes will be reviewed to ensure treatment quality and adherence. Treatment integrity will also be evaluated by means of randomly assessing recordings of treatment sessions against a session-by-session intervention checklist.

For the purpose of this study we will use an ERP protocol based on the inhibitory learning model of extinction [7], which states that the original fear conditioning is not erased during exposure therapy but stays intact while a second conditioning is developed. Translated to clinical practice, this means that during exposure and response prevention exercises, attention is focused on the disconfirmation of fear cognitions. Before exposure exercises, the fear cognitions are recorded and the exposure is introduced as a way to collect evidence for or against these appraisals. The ERP manual consists of three phases. In the first phase, an explanation of the behavioral model of OCD and rationale is discussed and an anxiety hierarchy containing all of the anxiety provoking situations is developed. The second phase includes both within-session and between-session in vivo and imaginal exposure-exercises, with early exposure to moderately distressing situations with progression toward more anxiety evoking ones. This latter is done to minimalize drop-out in the first exposure and response prevention sessions. Later, the patient is asked to practice in many different situations and circumstances and even alternate between the more easy and difficult exercises. According to the inhibitory learning model, the more variability is added throughout exposure exercises, the better the new information can be retrieved at a later point which minimize relapse [7]. Ritual prevention includes instructions to refrain from all compulsive behaviors. In the final phase, a relapse prevention plan is developed.

MCT focuses exclusively on modifying metacognitive beliefs about intrusive thoughts and the necessity of performing rituals [12]. MCT consists four treatment phases. Phase 1 involves psycho-education about the metacognitive model, increasing patients’ awareness of the role of metacognitions and generation of an idiosyncratic case conceptualization. This is accomplished by eliciting metacognitive beliefs, e.g. by guided questioning. Experiments are used to illustrate maladaptive coping strategies, e.g. the thought suppression experiment in which the patient is asked to suppress the thought of a white rabbit which is rarely completely successful [41]. Also, detached mindfulness (DM) is practiced. In DM, patients are asked to be aware of their intrusive thoughts and try to stop or disconnect any response to that thought, like engaging with their obsessional thoughts by worrying about consequences or the chance of occurrence [44]. Instead, patients practice with evaluating their intrusions and notice them as “just mental events in the mind,” e.g. by visualizing the thought moving away from them. In the second phase, metacognitive beliefs about intrusions are targeted by verbal cognitive restructuring (e.g. questioning the evidence and searching for counterevidence) and behavioral experiments. An example of such an experiment is exposure and response commission (ERC), in which patients are asked to perform rituals and to keep their intrusive thought in mind at the same time, instead of trying to get rid of the intrusive thought. The main aim of ERC is to enable patients to experience obsessive thoughts on a meta-level by obtaining distance from them and discovering that they are unimportant events in the mind [44]. In phase 3, metacognitive beliefs about rituals are challenged, again by means of both verbal methods (e.g. questioning the evidence and an advantages–disadvantages analysis) and behavioral experiments, such as ritual modulation experiments in which patients are asked to alternate between more and less ritual behavior with the aim of assessing its impact on daily life. In the final phase, the therapist and patient work on a relapse prevention plan consisting of a new plan for reacting in response to intrusive experiences combined with a blueprint of the therapy. The old versus new plan consists of attentional strategies and coping behaviors opposite to the strategies and behaviors of the old plan (e.g. applying detached mindfulness [new plan] instead of worrying about intrusions [old plan]). In addition, a blueprint of the therapy is developed, consisting of a summary of the therapy, the case conceptualization, a list of metacognitive beliefs and an overview of evidence challenging them. An overview of the both treatments is provided in Table 1. The full manuals (in Dutch) are available from the corresponding author upon request.

**Table 1:** Overview of metacognitive therapy (MCT; [39]; based on [44]) and exposure and response prevention (ERP) for obsessive-compulsive disorder (OCD)

Phase	MCT		ERP	
	Interventions	Sessions	Interventions	Sessions
1	- Provide treatment overview	1–2	- Provide treatment overview	1–3
	- Psycho-education about the metacognitive model of OCD		- Psycho-education about the behavioral model of OCD	
	- Elicit metacognitions by guided questioning		- Generation of a hierarchy of anxiety-provoking situations and avoidance behaviors	
	- Practicing of detached mindfulness			
2	- Modifying metacognitions about intrusions by verbal methods (e.g. questioning the evidence) and behavioral experiments (e.g. exposure with response commission, ritual postponement, and exposure and response prevention experiments)	3–8	- Exposure and response prevention exercises, both within-session and between sessions	4–13
3	- Modifying metacognitions about the necessity of rituals by verbal methods (e.g. questioning the evidence, advantages–disadvantages analysis of performing rituals) and behavioral experiments (e.g. ritual modulation experiments)	9–12	- Generation of a treatment summary consisting of an overview of OCD complaints pretreatment, rest symptoms at post-treatment, and a relapse prevention plan containing helpful interventions to maintain	14–15
4	Generation of a new plan for processing in response to unwanted thoughts, feelings, or events and a therapy blueprint consisting of the case conceptualization, a list of metacognitive beliefs and an overview of evidence challenging them	13–15		

### Statistical analysis

#### Outcomes

Data will be analyzed using SPSS for Windows version 25.

Because of the expected drop-out and the uneven time intervals between measurements (post-test 6-month–30-month follow-up), the use of mixed models is the most appropriate statistical method [36]. This methodology is very suitable to analyze repeated measures by taking dependency between observation into account and the ability to handle missing data. In case of Missing At Random (MAR), we will use these variable(s) as covariate in our analysis. In case of Missing Not At Random (MNAR) we will use pattern mixture models. Mixed models will be adjusted by the baseline values of the repeated measures. Descriptives for means and proportions of baseline clinical and demographic variables between treatment conditions will be reported so potential magnitudes of imbalances can be assessed. Model diagnostics will be assessed by exploring residual plots. In case they are not acceptable, we will apply bootstrapping procedures with the use of R (R core team, 2018).

Fixed effects in our model will be time, treatment, and their interaction. In case of missingness, we will add these variable(s) also as fixed effects. The Benjamini–Hochberg procedure is applied to the significances of the time*treatment interaction *p*-values (two-sided *p* < 0.05) of the different outcome measures. The time variable will be treated categorically, with the first post-baseline measurement as the reference category. To accommodate the modeling of correlation among repeated measurements, we impose a first order autoregressive (AR (1)) structure on the residuals. Next, the interaction effect between time and group will be explored by analyzing the estimated marginal means at different time points. We expect a significant interaction effect between time and group, which means that scores change differently over time in the two treatment conditions. More specifically, we expect a more negative time trend for MCT than for ERP, indicating that the measurement scores in the MCT condition decline more over time than in the ERP condition. To gain further insight into the statistical significance of the improvements achieved in the two treatment conditions, we will perform a least significant difference test with the estimated marginal means to compare changes between treatment conditions. In accordance with the linear mixed models, we expect a statistically significant decline in both treatment conditions between pretest and post-test, but no statistical differences between post-test and both follow-up measures. To allow for comparison with other studies into the effectiveness of ERP and MCT for OCD, Cohen’s *d* statistic ((mean 1 – mean 2) / pooled SD) will be employed to calculate within-group effect sizes (ES) for changes on outcome measures and to evaluate between-group differences. We will calculate Cohen’s *d* statistics for both intent-to-treat samples by using multilevel analysis with all available data and completer samples (our primary analysis: minimum of eight treatment sessions and no change in medication during treatment will be managed as criteria for each patient to can be classified as treatment completer). Based on previous research, we expect a large within-treatment ES for both treatment conditions (Cohen’s *d* > 0.8). We expect a medium between-group ES in favor of MCT. In addition, the clinical significance of treatment effects and amount of drop-out will be examined also to gain further insight into the clinical value of the two treatment conditions.

#### Endpoints

The clinical significance of treatment effects will be examined using the procedures outlined by Jacobson and Truax [21]. Patients will be classified as recovered, if they score within the normal range on the Y-BOCS after treatment (cut-off point = 14) and display statistically reliable improvement on that measure (reliable change index = 10) [21]. Patients will be classified as improved but not recovered if they meet only one criterion. Patients will be classified as asymptomatic (a more stringent criterion for defining recovery) when they achieve a posttreatment score of ≤ 7 (indicating an almost total absence of OCD symptomatology), in addition to meeting the reliable change index. Further, diagnosis-free status will also be used as an index of clinically significant change.

## Discussion

MCT is a relatively new treatment for OCD, based on a metacognitive model that states that, rather than the intrusive thoughts and compulsive behaviors, it is in fact beliefs about the meaning and significance of obsessive thoughts on the one hand and beliefs about the need to conduct rituals and neutralizing behaviors on the other hand that are crucial for the development of OCD As a result, interventions should be targeted at these metacognitive beliefs. Our hypothesis is that MCT is more efficacious in the treatment of OCD than the current “gold standard” psychological treatment for OCD, ERP. Since there is a wide variation in symptomatology between OCD patients, and beliefs about intrusions and compulsions are comparable for each subtype, it may be that MCT is particularly well suited in the treatment of this disorder. Moreover, it may be that MCT is less burdensome since it does not include prolonged exposure to anxiety provoking stimuli. So far, five relatively small studies suggest that MCT might be an efficacious treatment for OCD and may be even more efficacious than the current “gold standard,” ERP [12, 16, 33, 35, 39]. We presented the rationale and design of a RCT assessing the relative efficacy of ERP and MCT for OCD. To our knowledge, this is the first long-term RCT to explore whether MCT produces better results than ERP.

The study has several strengths, including randomization of patients to two active treatment conditions, use of an unselected, clinically representative sample of OCD patients, and long-termfollow-up assessments.

However, the current study also has limitations. Treatment conditions might be contaminated as participating therapists will deliver both ERP and MCT. It might also be difficult to maintain treatment integrity as both treatments will be conducted by therapists who work within a routine outpatient community mental health center. We aim to minimize these limitations by means of reviewing all active cases in consultation meetings and careful checking of treatment integrity.

### Trial status

This study received ethical approval from the Medical Ethical Committee of the Leiden University Medical Centre (LUMC) on 21 October 2014. The first patient enrolled on 6 February 2015. Sixty-eight participants are randomized so far. We are still recruiting patients and have planned to close the inclusion at the end of 2019.

## Additional file

Additional file 1: SPIRIT 2013 Checklist: Recommended items to address in a clinical trial protocol and related documents. (DOC 254 kb)

## Abbreviations

AR (1): First order autoregressive
BARI: Beliefs About Rituals Inventory
BDI: Beck Depression Inventory
CBT: Cognitive behavioral therapy
CT: Cognitive therapy
DM: Detached mindfulness
DSM: Diagnostic and Statistical Manual of Mental Disorders
ERP: Exposure and response prevention
ES: Effect size
LUMC: Leiden University Medical Centre
MAR: Missing at Random
MCT: Metacognitive therapy
MNAR: Missing Not at Random
OBQ: Obsessive Belief Questionnaire
OCD: Obsessive compulsive disorder
Padua-IR: Padua Inventory-Revised
RCT: Randomized controlled trial
SCID-I: Structured Clinical Interview for DSM Axis I Disorders
SCL-90: Symptom Checklist
SD: Standard deviation
SPIRIT: Standard Protocol Items: Recommendations of Interventional Study’s
SPSS: Statistical Package for the Social Sciences
SSRI: Selective serotonin reuptake inhibitors
TAF: Thought action fusion
TCRF: Treatment Change Recording Form
TEF: Thought event fusion
TFI: Thought fusion instrument
TOF: Thought object fusion
WHOQOL: World Health Organization Quality of Life
Y-BOCS: Yale-Brown Obsessive Compulsive Scale

## References

[1] American Psychiatric Association (2013). Diagnostic and Statistical Manual of Mental Disorders (5th edition (DSM-5)).

[2] Beck AT, Steer RA, Brown GK (1996). Manual for the Beck Depression Inventory-II.

[3] Blanco C, Olfson M, Stein DJ, Simpson HB, Gameroff MJ, Narrow MH (2006). Treatment of obsessive–compulsive disorder by U.S. psychiatrists. J Clin Psychiatry.

[4] Burns GL, Keortge S, Formea G, Sternberger L (1996). Revision of the Padua Inventory of obsessive-compulsive disorder symptoms: Distinctions between worry, obsessions, and compulsions. Behav Res Ther.

[5] Clark DA (2004). Cognitive-behavioral therapy for OCD.

[6] Cohen JA (1992). Power primer. Psychol Bull.

[7] Craske MG, Treanor M, Conway C, Zbozinek T, Vervliet B (2016). Maximizing Exposure Therapy: An inhibitory learning approach. Behav Res Ther.

[8] Derogatis LR (1983). SCL-90-R: administration, scoring & procedures: Manual II.

[9] Eddy KT, Dutra L, Bradely R, Westen DA (2004). Multidimensional meta-analysis of psychotherapy and pharmacotherapy for obsessive-compulsive disorder. Clin Psychol Rev.

[10] First MB, Spitzer RL, Gibbon M, Williams JBW. Structured Clinical Interview for DSM-IV-TR Axis-I Disorders (Research Version, Patient Edition (SCID-I/P). New York: Biometrics Research Department, NY State Psychiatric Institute; 2001.

[11] Fisher PL, Wells A (2005). How effective are cognitive and behavioral treatments for obsessive-compulsive disorder: A clinical significance analysis. Behav Res Ther.

[12] Fisher PL, Wells A (2008). Metacognitive therapy for obsessive-compulsive disorder: A case series. J Behav Ther Exp Psychiatry.

[13] Fisher PL (2009). Obsessive Compulsive Disorder: A comparison between CBT and the metacognitive approach. Int J Cogn Ther.

[14] Fisher PL, Wells A (2005). How effective are cognitive and behavioral treatments for obsessive-compulsive disorder: a clinical significance analysis. Behav Res Ther.

[15] Fisher P, Wells A (2009). Metacognitive therapy.

[16] Fitt S, Rees C (2012). Metacognitive Therapy for obsessive compulsive disorder by videoconference: A preliminary study. Behav Change.

[17] Frost RO, Steketee G (2002). Cognitive approaches to obsessions and compulsions: Theory, assessment, and treatment.

[18] Frost RO, Steketee G, Krause MS, Trepanier KL (2010). The relationship of the Yale-Brown Obsessive Compulsive Scale (YBOCS) to other measures of obsessive compulsive symptoms in a nonclinical population. J Pers Assess.

[19] Goodman WK, Price LM, Rasmussen SA, Mazure C, Fleischmann RL, Hill CL (1995). The Yale-Brown Obsessive Compulsive Scale (Y-BOCS): Part I. Development, use, and reliability. Arch Gen Psychiatry.

[20] Gwilliam PDH, Wells A, Cartwright-Hatton S (2004). Does metacognition or responsibility predict obsessive–compulsive symptoms: A test of the metacognitive model. Clin Psychol Psychother.

[21] Jacobson NS, Truax P (1991). Clinical significance: A statistical approach to defining meaningful change in psychotherapy research. J Consul Clin Psychol.

[22] Liu G, Liang KY (1997). Sample size calculations for studies with correlated observations. Biometrics.

[23] Lobbestael J, Leurgans M, Arntz A (2010). Inter-rater reliability of the Structured Clinical Interview for DSM-IV Axis I Disorders (SCID I) and Axis II Disorders (SCID II). Clin Psychol Psychother.

[24] McLeod AI (1985). Remark AS R58. A remark on algorithm AS 183. An efficient and portable pseudo-random number generator. Appl Stat.

[25] McNicol K, Wells A (2012). Metacognitions and obsessive-compulsive symptoms: the contribution of thought-fusion beliefs and beliefs about rituals. Int J Cogn Ther.

[26] Meyer V (1966). Modifications of expectations in case of obsessional rituals. Behav Res Ther.

[27] Mowrer OH (1951). Two-factor learning theory: summary and content. Psychological Review.

[28] Myers SG, Fisher PL, Wells A (2008). Belief domains of the Obsessive Beliefs Questionnaire-44 (OBQ-44) and their specific relationship with obsessive– compulsive symptoms. J Anxiety Disord.

[29] Obsessive Compulsive Cognitions Working Group (2005). Psychometric validation of the Obsessive Beliefs Questionnaire and the Interpretation of Intrusions Inventory: Part 2: Factor analyses and testing of a brief version. Behav Res Ther.

[30] Olatunji BO, Cisler JM, Deacon BJ (2010). Efficacy of cognitive behavioral therapy for anxiety disorders: a review of meta-analytic findings. Psychiatr Clin North Am.

[31] Ost LG, Havnen A, Hansen B, Kvale G (2015). Cognitive behavioral treatments of obsessive-compulsive disorder. A systematic review and meta- analysis of studies published 1993–2014. Clin Psychol Rev.

[32] Rachman S (1993). Obsessions, responsibility and guilt. Behav Res Ther.

[33] Rees CS, van Koesveld KE (2008). An open trial of group metacognitive therapy for obsessive-compulsive disorder. J Behav Ther Exp Psychiatry.

[34] Rosa-Alcazar AI, Sanchez-Meca J, Gomez-Conesa A, Martin-Martinez F (2008). Psychological treatment of obsessive-compulsive disorder: A meta-analysis. Clin Psychol Rev.

[35] Simons M, Schneider S, Herpertz-Dahlmann B (2006). Metacognitive therapy versus exposure and response prevention for pediatric obsessive-compulsive disorder. Psychother Psychosom.

[36] Singer JD, Willett JB (2003). Applied longitudinal data analysis: modelling change and event occurrence.

[37] Skapinakis P, Caldwell D, Hollingworth W, Bryden P, Fineberg N, Salkovskis P (2016). A systematic review of the clinical effectiveness and cost-effectiveness of pharmacological and psychological interventions for the management of obsessive-compulsive disorder in children/adolescents and adults. Health Technol Assess.

[38] Tolin DF, Maltby N, Diefenbach GJ, Hannan SE, Worhunsky P (2004). Cognitive-behavioral therapy for medication non-responders with obsessive compulsive disorder: a wait-list-controlled open trial. J Clin Psychiatry.

[39] van der Heiden C, Rossen van K, Dekker A, Damstra M, Deen M (2016). Metacognitive therapy for obsessive-compulsive disorder: A pilot study. J Obsessive Compulsive Relat Disord.

[40] van Oppen P, Emmelkamp PMG, Balkom van AJLM, van Dyck R (1995). The sensitivity to change of measures for obsessive-compulsive disorder. J Anxiety Disord.

[41] Wegner DM, Schneider DJ, Carter SR, White TL (1987). Paradoxical effects of thoughts suppression. J Pers Soc Psychol.

[42] Wells A (1997). Cognitive therapy of anxiety disorders: a practice manual and conceptual guide.

[43] Wells A (2000). Emotional disorders and metacognition: innovative cognitive therapy.

[44] Wells A (2009). Metacognitive therapy for anxiety and depression.

[45] Wells A, Gwilliam P, Cartwright-Hatton S (2001). The Thought Fusion Instrument.

[46] Whittal ML, Thordarson PD, McLean PD (2005). Treatment of obsessive-compulsive disorder: Cognitive behavior therapy vs. exposure and response prevention. Behav Res Ther.

[47] Whittal ML, Robichaud M, Thordarson DS, McLean PD (2008). Group and individual treatment of obsessive-compulsive disorder using cognitive therapy and exposure plus response prevention: a 2-year follow-up of two randomized trials. J Consult Clinical Psychol.

[48] World Health Organization (2004). The World Health Organization Quality Of Life (WHOQOL) –Bref.

